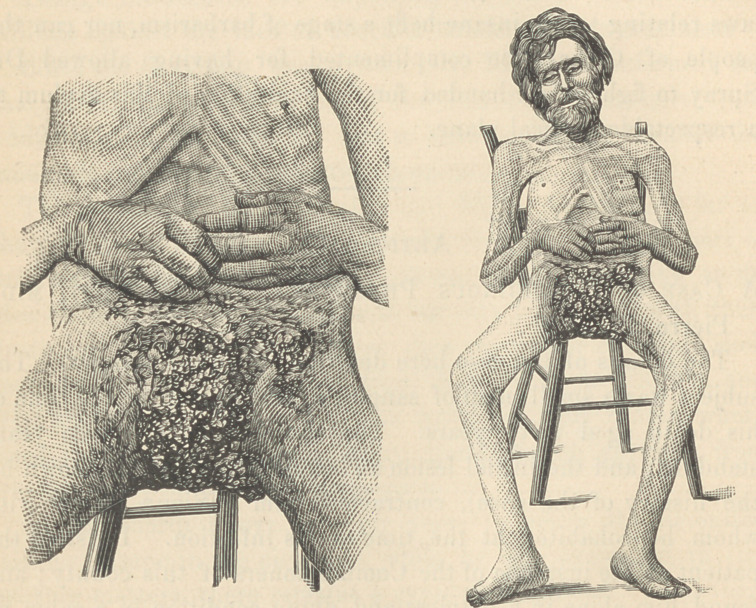# A Case of Serpiginous Phagedena

**Published:** 1883-11

**Authors:** D. Scott

**Affiliations:** Pierre, D. T.


ARTICLE II.

A CASE OF SERPIGINOUS PHAGEDENA. By D. SCOTT, M.D., Pierre, D. T.

The details of the case here described are quite meager. The subject was a small man of sanguine temperment, at the time of his death aged forty years. The disease was of three years standing; and the initial lesion an infecting chancre (as shown by the history of the case), contracted from a Sioux squaw with whom he cohabited at the time of his infection. I visited the patient at the instance of the Commissioners of this county; and found him alone in a helpless and dying condition in a cabin on the Missouri river bottom, eighteen miles above this city. He was too feeble to give me any details of his horrible case. Lying prone upon the floor without any covering save a filthy blanket, deserted by his one neighbor, tormented by flies and eaten by maggots, he presented a spectacle to be seen nowhere save on the confines of civilization.

When I stooped to remove the filthy rags that covered the ulcer, the poor wretch with a yet lingering shame uttered a feeble protest, but off they came and for the first time my stomach, fortified by an experience of nearly twenty years, threatened to rebel. Under the circumstances what was the duty of the conscientious physician ? Down through the ages I heard sounding the injunction of Confucius and of Christ: "As ye would that others should do unto you, do ye even so unto them." In the application of this golden rule, as I interpreted it, I gave him a teaspoonful of Magendie's solution of morphia which carried him rapidly into a peaceful oblivion and final *euthanasia* twelve hours later, as I learned from his solitary neighbor next day. The photographs do not show fully the details of the ravages of the disease. The ulcer had burrowed down over the perinæum and up over the sacrum. The penis, testicles and in fact every vestige of the external genitalia had disappeared except traces of the spermatic cords. The testes probably perished from secondary inflammation after being divested of their natural coverings. The only light I could get on the early history of the case was kindly furnished me from the records of the Military Hospital at Fort Sully by Dr. Lauderdale, Surgeon of the 11th Infantry at that place. From these it appeared that the subject was admitted for treatment July, 1877, "with syphilitic ulcers of penis." "Penis amputated and patient discharged with wound nearly healed

Sept., 1879." Dr. Landerdale not having charge of the hospital at that time, had no personal knowledge of the case. This much is known, bowever, that he never received any regular treatment afterward. The post mortem showed that the bones had suffered extensively from tertiary lesions, notably the long bones of the lower extremity. As shown in the photographs, the tibiæ were much enlarged and nodulated by periostitis and exostoses, all of ancient date, however. No active lesion was visible except an open gummatous ulcier on the outside of one ankle involving the malleolus. I have dwelt on the presence of syphilis in this case both because the death was not wholly due to the phagedena and also because of the rarity of the two in association. Phagedena in both its forms usually accompanies chancroid and not chancrous sores. Incidentally let me add that phagedena is a very common complication of venereal sores among Indians and also among white men who cohabit with them.

In the hope that this brief history may afford some interest to your readers, even though as a matter of fact it contain nothing new, I have ventured to describe in this brief way its special features.

## Figures and Tables

**Figure f1:**